# Prevalence of low birth weight and macrosomia estimates based on heaping adjustment method in China

**DOI:** 10.1038/s41598-021-94375-2

**Published:** 2021-07-22

**Authors:** Liping Shen, Jie Wang, Yifan Duan, Zhenyu Yang

**Affiliations:** National Institute for Nutrition and Health, Chinese Center for Disease Control and Prevention, Nanwei road No.29, Xicheng district, Beijing, 100050 China

**Keywords:** Malnutrition, Obesity, Nutrition

## Abstract

Low birth weight (< 2500 g; LBW) and macrosomia (> 4000 g) are both adverse birth outcomes with high health risk in short- or long-term period. However, national prevalence estimates of LBW and macrosomia varied partially due to methodology limits in China. The aim of this study is to estimate the prevalence of LBW and macrosomia after taking potential birth weight heaping into consideration in Chinese children under 6 years in 2013. The data were from a nationally representative cross-sectional survey in mainland China in 2013, which consists of 32,276 eligible records. Birth weight data and socio-demographic information was collected using standard questionnaires. Birth weight distributions were examined and LBW and macrosomia estimates were adjusted for potential heaping. The overall prevalence of LBW of Chinese children younger than 6 years was 5.15% in 2013, with 4.57% in boys and 5.68% in girls. LBW rate was higher for children who were minority ethnicity, had less educated mothers, mothers aged over 35 years or under 20 years, or were in lower income household than their counterparts. The overall prevalence of macrosomia of Chinese children younger than 6 years was 7.35% in 2013, with 8.85% in boys and 5.71% in girls. The prevalence of macrosomia increased with increasing maternal age, educational level and household income level. Both LBW and macrosomia varied among different regions and socio-economic groups around China. It is found that estimates based on distribution adjustment might be more accurate and could be used as the foundation for policy-decision and health resource allocation. It would be needed to take potential misclassification of birth weight data arising from heaping into account in future studies.

## Introduction

Birth weight is a strong predictor for mortality and morbidity during infancy and later life. Both low birth weight and macrosomia are great concerns to public health. LBW is thought to place a high risk on children survival, growth, development and has long-term impact on health outcomes such as diabetes and cardiovascular diseases in later life^1^. Globally, the prevalence of LBW was 14.6% and about 20.5 million babies were born with LBW in 2015, of which 91.0% were in developing countries^2^. Macrosomia was associated with increased risks of infant morbidity, and obesity and metabolic complications in childhood and adulthood^3–5^. The prevalence of macrosomia ranged from 5 to 20% in developed countries and from 0.5 to 14.9% in developing countries, while an overall 15–25% increase around the world was also found in the past two to three decades^6,7^.

The prevalence of LBW and macrosomia in China varied across different studies in the past two decades. For example, a study of 18,554 livebirths from 14 provinces found a LBW rate of 4.6% in 2006^8^. Another study reported a LBW rate of 2.87% among 16,954 infants in 5 provinces around China between 2006 and 2008^9^. A 39-hospital based study showed a LBW rate of 6.1% in 14 provinces of China (n = 101,163) in 2011^10^. China’s National Maternal Near Miss Surveillance System (NMNMSS) of 441 health facilities indicated a LBW rate of 5.36% in China between 2012 and 2014^11^. The prevalence of macrosomia varied across different studies in China too. Perinatal Health Care Surveillance System in 12 cities and counties in southeast China showed the macrosomia rate of 7.83% in 2005^12^ and a cross-sectional study of 14 provinces in China reported a macrosomia rate of 6.5% in 2006^13^. Another studies reported a macrosomia rate of 10.44% in 2006–2008^9^, and 7.3% in 2011^14^. Halves of these studies were health facility-based studies and the other were community-based studies.

It is found that when information on birth weight is collected from either health facility records or self-reported by mothers or other caregivers, the data tend to heap around multiples of 500 g^15^. As a result, a certain proportion of infants whose birth weight are exactly 2500 g (the cut-off point for LBW) actually < 2500 g or > 2500 g and those are exactly 4000 g (the cut-off point for macrosomia) actually > 4000 g or < 4000 g. This could dramatically misestimates the prevalence of LBW and macrosomia^16^. However, most LBW and macrosomia estimates did not take potential misclassification of birth weight data arising from heaping into account. A modified and reliable estimate of LBW and macrosomia is needed to ensure accuracy at the national and international levels to guide future interventions and policies.

The purpose of this study was to estimate the prevalence of LBW and macrosomia by geographic areas, age and socio-demographic accounting for potential birth weight heaping, using a representative survey conducted in China in 2013.

## Results

### Description of the study population

**Figure 1:**
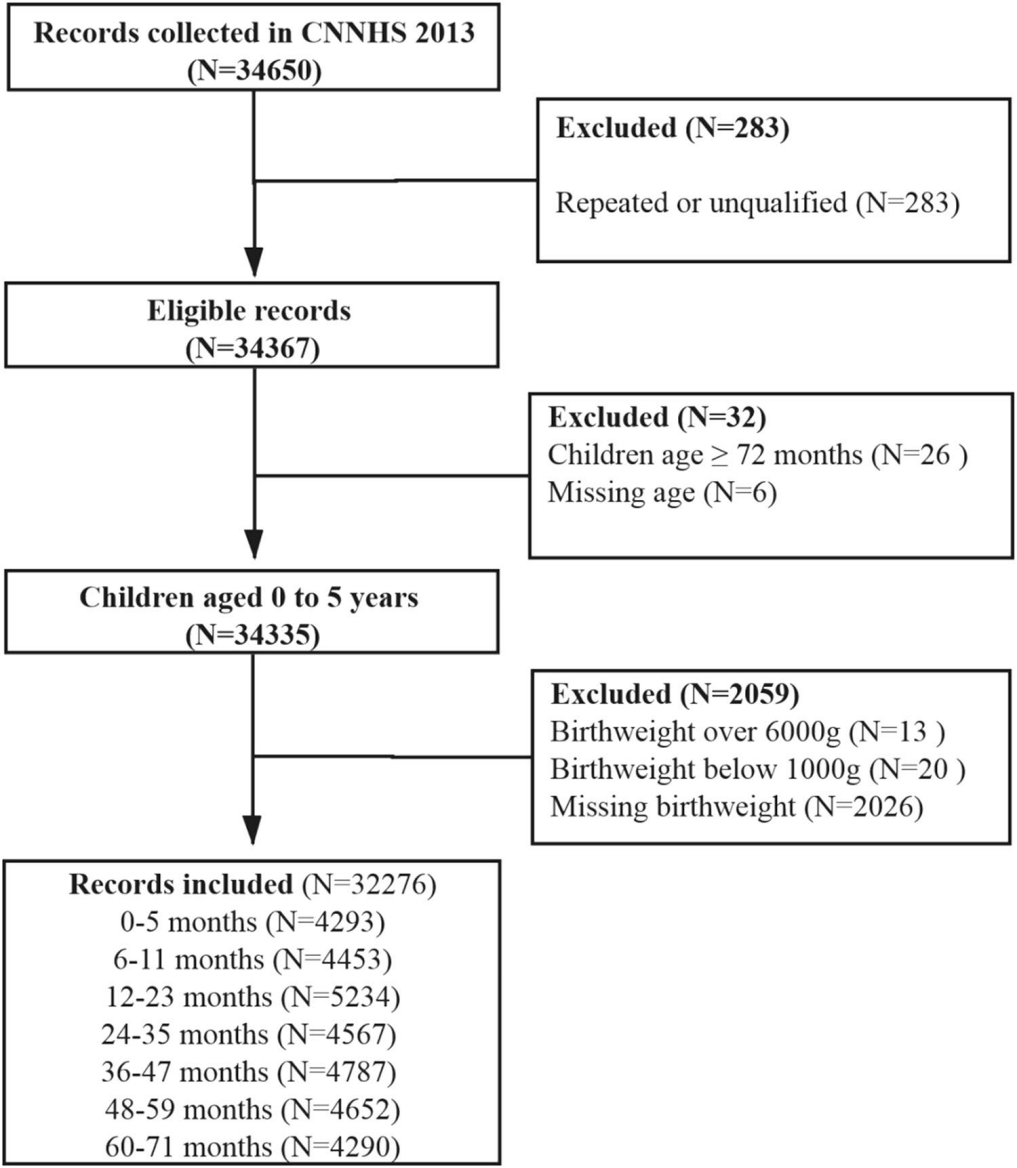
Flow of study participants.

Overall, 32,276 records were included in the analysis (Fig. 1, Table 1). The characteristics of the subjects included and excluded in the analysis were shown in Supplementary Table 1. Two groups were similar in gender and maternal age. More children aged 3–5 years, minority ethnicity, with a lower educated mother, from lower income household or rural areas were seen in the excluded group. The mean birth weight among children under 6 years was 3294.9 ± 484.8 g. Birth weight was greater for boy than for girl (3335.6 ± 491.3 vs 3251.9 ± 474.0, t = 15.6, P < 0.0001). Maternal age ranged from 15 to 50 years, with a mean age of 29.2 ± 5.3 years.

**Table 1:** Demographic and socioeconomic characteristics of the subjects.

Characteristic	N (%) or mean ± SD
Birth weight	3294.9 ± 484.8 g
**Age groups**	
0–11 months	27.1% (8746/32,276)
12–23 months	16.2% (5234/32,276)
24–35 months	14.2% (4567/32,276)
36–47 months	14.8% (4787/32,276)
48–59 months	14.4% (4652/32,276)
60–71 months	13.3% (4290/32,276)
**Gender**	
Girl	48.6% (15,680/32,276)
Boy	51.4% (16,596/32,276)
**Residential area**	
Urban-metropolis	22.6% (7299/32,276)
Urban-middle or small cities	28.6% (9220/32,276)
Rural-non-poor areas	32.7% (10,565/32,276)
Rural-poor areas	16.1% (5192/32,276)
**Ethnicity**^a^	
Han	86.9% (28,037/32,267)
Minority	13.1% (4230/32,267)
**Maternal age groups**^b^	
< 20 years	1.2% (355/30,421)
20–25 years	25.1% (7627/30,421)
26–30 years	37.2% (11,321/30,421)
31–35 years	24.1% (7333/30,421)
> 35 years	12.4% (3785/30,421)
**Maternal education**^c^	
Primary or below	12.6% (3841/30,599)
Junior high school	47.2% (14,450/30,599)
High school	18.2% (5569/30,599)
Associate's degree	11.1% (3392/30,599)
Bachelor's degree or above	10.9% (3347/30,599)
**Annual household income (per capital CNY)**^d^	
< 10,000	34.8% (11,242/32,261)
10,000–19,999	27.6% (8888/32,261)
≥ 20,000	27.5% (8880/32,261)
Refuse to response	10.1% (3251/32,261)

^a^The ethnic information for 9 subjects was missing.

^b^Information on maternal age was missing in 1855 subjects.

^c^Information on the maternal educational level was missing in 1677 subjects.

^d^Information on the annual household income was missing in 15 subjects.

### Low birth weight prevalence

The overall prevalence of low birth weight of Chinese children younger than 6 years was 5.15% (95% CI 5.01–5.33%) in 2013, with 4.57% (95% CI 4.39–4.80%) in boys and 5.68% (95% CI 5.41–5.98%) in girls (Table 2). The prevalence of low birth weight ranged from 3.75% (95% CI 3.52–4.01%) in central China to 7.34% (95% CI 6.95–7.72%) in west China, and from 4.75% (95% CI 4.55–4.99%) in urban areas to 5.57% (95% CI 5.36–5.86%) in rural areas. The prevalence of low birth weight was higher in minority ethnicity (7.52%, 6.94–8.11%) than Han ethnicity (4.83%, 4.66–5.00%). There was a higher percentage of LBW for children born to mothers who were under 20 years (8.84%, 6.77–11.39%) and over 35 years (5.93%, 5.42–6.48%), compared to 20–25 years mothers (5.19%, 4.81–5.52%). The prevalence of low birth weight in children was nearly twice higher for their mothers with primary or below education level than those mothers with a bachelor's degree or above (7.24%, 6.69–7.97% vs 3.65%, 3.26–4.14%). The prevalence of low birth weight decreased from 6.42% (95% CI 6.08–6.77%) in children from < 10,000 income household to 4.18% (95% CI 3.92–4.55%) in children from ≥ 20,000 income household.

**Table 2:** The prevalence of LBW among Chinese children under 6 years in 2013.

Characteristics	Total			Boys			Girls		
	Total Number	%	95% CI	Total Number	%	95% CI	Total Number	%	95% CI
Total^a^	31,969	5.15	5.01, 5.33	16,439	4.57	4.39, 4.80	15,530	5.68	5.41, 5.98
**Age groups (months)**									
0–11	8682	4.78	4.47, 5.07	4436	4.15	3.76, 4.56	4246	5.33	4.87, 5.81
12–23	5172	5.27	4.85, 5.73	2704	4.85	4.33, 5.46	2468	5.62	5.00, 6.30
24–35	4515	5.48	5.04, 5.97	2326	4.88	4.31, 5.51	2189	6.08	5.31, 6.81
36–47	4748	5.34	4.90, 5.82	2454	4.48	3.96, 5.15	2294	6.29	5.52, 6.99
48–59	4599	5.29	4.84, 5.77	2357	4.79	4.25, 5.50	2242	5.75	5.12, 6.44
60–71	4253	5.18	4.64, 5.67	2162	4.69	4.06, 5.33	2091	5.49	4.84, 6.29
**Residential area**									
East China	11,600	4.60	4.32, 4.88	5918	4.08	3.79, 4.45	5682	5.15	4.73, 5.60
Central China	10,054	3.75	3.52, 4.01	5193	3.35	3.03, 3.69	4861	4.13	3.77, 4.56
West China	10,315	7.34	6.95, 7.72	5328	6.58	6.07, 7.01	4987	8.02	7.44, 8.64
**Urban**									
Subtotal	16,421	4.75	4.55, 4.99	8361	4.20	3.91, 4.54	8060	5.22	4.91, 5.64
Large cities	7256	4.14	3.80, 4.42	3666	3.57	3.17, 3.99	3590	4.60	4.16, 5.14
Medium and small cities	9165	5.28	4.97, 5.63	4695	4.76	4.35, 5.21	4470	5.80	5.33, 6.32
**Rural**									
Subtotal	15,548	5.57	5.36, 5.86	8078	5.00	4.69, 5.32	7470	6.21	5.75, 6.58
Non-poor rural areas	10,451	4.85	4.57, 5.15	5398	4.26	3.95, 4.67	5053	5.44	4.98, 5.91
Poor rural areas	5097	6.61	6.68, 7.58	2680	6.53	5.90, 7.23	2417	7.77	7.06, 8.69
**Ethnicity**									
Han	27,828	4.83	4.66, 5.00	14,238	4.24	3.99, 4.48	13,590	5.43	5.15, 5.64
Minority	4132	7.52	6.94, 8.11	2196	7.14	6.38, 7.98	1936	7.76	6.98, 8.77
**Maternal age groups**									
< 20 years	351	8.84	6.77, 11.39	178	7.61	5.20, 11.04	173	10.24	7.01, 14.45
20–25 years	7558	5.19	4.81, 5.52	3880	4.91	4.41, 5.39	3678	5.40	4.92, 6.00
26–30 years	11,213	4.59	4.33, 4.87	5914	3.94	3.66, 4.30	5299	5.19	4.77, 5.63
31–35 years	7278	4.63	4.25, 4.94	3727	3.99	3.60, 4.47	3551	5.17	4.71, 5.78
> 35 years	3752	5.93	5.42, 6.48	1974	5.11	4.47, 5.85	1778	6.79	5.95, 7.72
**Maternal education**									
Primary or below	3758	7.24	6.69, 7.97	2048	6.17	5.52, 7.00	1710	8.46	7.46, 9.53
Junior high school	14,331	5.07	4.86, 5.34	7479	4.62	4.27, 4.93	6852	5.54	5.21, 5.99
High school	5535	4.43	4.12, 4.83	2885	3.78	3.34, 4.23	2650	5.20	4.63, 5.84
Associate's degree	3372	4.44	3.95, 4.94	1661	4.26	3.63, 4.99	1711	4.48	3.83, 5.20
Bachelor's degree or above	3331	3.65	3.26, 4.14	1686	3.10	2.58, 3.68	1645	4.30	3.66, 5.02
**Annual household income (per capital CNY)**^**b**^									
< 10,000	11,102	6.42	6.08, 6.77	5759	5.70	5.26, 6.07	5343	7.11	6.70, 7.68
10,000–19,999	8815	4.60	4.25, 4.92	4550	4.25	3.85, 4.67	4265	4.84	4.42, 5.32
≥ 20,000	8819	4.18	3.92, 4.55	4491	3.68	3.34, 4.07	4328	4.69	4.27, 5.15

^a^Information on preterm was missing for 307 subjects.

^b^3251 of subjects refused to answer the question about annual household income.

### Macrosomia prevalence

The overall prevalence of macrosomia of Chinese children younger than 6 years was 7.35% (95% CI 7.08–7.49%) in 2013, with 8.85% (95% CI 8.53–9.01%) in boys and 5.71% (95% CI 5.59–5.94%) in girls (Table 3). The prevalence of macrosomia ranged from 6.06% (95% CI 5.82–6.30%) in west China to 8.08% (95% CI 7.78–8.38%) in east China, and from 6.81% (95% CI 6.55–6.94%) in rural areas to 7.78% (95% CI 7.64–8.08%) in urban areas. The prevalence of macrosomia was higher in Han ethnicity (7.49%, 7.35–7.64%) than Minority ethnicity (5.59%, 5.26–5.94%). The prevalence of macrosomia increased with increasing maternal age, educational level and household income level (Table 3).

**Table 3:** The prevalence of macrosomia among Chinese children under 6 years in 2013.

Characteristics	Total			Boys			Girls		
	Total number	%	95% CI	Total number	%	95% CI	Total number	%	95% CI
Total	32,276	7.35	7.08, 7.49	16,596	8.85	8.53, 9.01	15,680	5.71	5.59, 5.94
**Age groups (months)**									
0–11	8746	7.78	7.49, 8.08	4467	9.18	8.69, 9.68	4279	6.30	5.94, 6.68
12–23	5234	8.08	7.64, 8.53	2735	9.51	8.85, 10.20	2499	6.55	6.06, 7.08
24–35	4567	6.81	6.43, 7.21	2355	8.08	7.49, 8.69	2212	5.37	4.95, 5.82
36–47	4787	6.55	6.18, 6.94	2469	8.08	7.49, 8.69	2318	4.85	4.46, 5.26
48–59	4652	7.08	6.68, 7.49	2388	8.53	7.93, 9.18	2264	5.59	5.16, 6.06
60–71	4290	7.08	6.68, 7.35	2182	9.18	8.38, 9.85	2108	4.95	4.55, 5.37
**Residential area**									
East China	11,697	8.08	7.78, 8.38	5964	9.68	9.34, 10.20	5733	6.55	6.18, 6.81
Central China	10,127	7.49	7.21, 7.78	5223	8.85	8.53, 9.34	4904	5.94	5.59, 6.30
West China	10,452	6.06	5.82, 6.30	5409	7.49	7.21, 7.93	5043	4.55	4.27, 4.75
**Urban**									
Subtotal	16,519	7.78	7.64, 8.08	8408	9.68	9.18, 10.03	8111	5.94	5.71, 6.18
Large cities	7299	8.23	7.93, 8.69	3685	10.03	9.51, 10.56	3614	6.55	6.06, 6.94
Medium and small cities	9220	7.35	7.08, 7.64	4723	9.34	8.85, 9.68	4497	5.48	5.16, 5.82
**Rural**									
Subtotal	15,757	6.81	6.55, 6.94	8188	7.93	7.64, 8.38	7569	5.48	5.26, 5.71
Non-poor rural areas	10,565	6.18	5.94, 6.43	5451	7.49	7.08, 7.93	5114	4.85	4.55, 5.16
Poor rural areas	5192	7.78	7.35, 8.23	2737	8.85	8.38, 9.51	2455	6.55	6.18, 7.08
**Ethnicity**									
Han	28,037	7.49	7.35, 7.64	14,340	9.01	8.85, 9.34	13,697	5.94	5.71, 6.18
Minority	4230	5.59	5.26, 5.94	2251	7.08	6.43, 7.64	1979	4.09	3.67, 4.46
**Maternal age groups**									
< 20 years	355	5.37	4.36, 6.68	181	6.18	4.65, 8.23	174	4.65	3.36, 6.30
20–25 years	7627	6.18	5.82, 6.43	3915	7.21	6.81, 7.64	3712	5.05	4.65, 5.37
26–30 years	11,321	7.08	6.81, 7.35	5974	8.69	8.23, 9.01	5347	5.37	5.05, 5.59
31–35 years	7333	8.08	7.78, 8.53	3753	9.34	8.85, 10.03	3580	6.81	6.30, 7.21
> 35 years	3785	9.68	9.01, 10.20	1989	12.10	11.12, 12.92	1796	6.94	6.43, 7.64
**Maternal education**									
Primary or below	3841	5.82	5.48, 6.18	2089	6.94	6.30, 7.49	1752	4.65	4.18, 5.05
Junior high school	14,450	7.21	6.94, 7.49	7542	8.85	8.53, 9.34	6908	5.48	5.16, 5.71
High school	5569	7.49	7.08, 7.93	2902	8.53	8.08, 9.18	2667	6.30	5.94, 6.81
Associate's degree	3392	8.38	7.93, 9.01	1670	10.56	9.68, 11.51	1722	6.43	5.82, 7.08
Bachelor's degree or above	3347	8.53	7.93, 9.01	1696	10.03	9.18, 10.93	1651	6.94	6.30, 7.64
**Annual household income (per capital CNY)**^**a**^									
< 10,000	11,242	7.08	6.81, 7.35	5829	8.38	7.93, 8.69	5413	5.82	5.48, 6.18
10,000–19,999	8888	7.21	6.81, 7.49	4585	8.85	8.38, 9.34	4303	5.37	5.05, 5.71
≥ 20,000	8880	7.78	7.49, 8.23	4531	9.68	9.18, 10.20	4349	6.06	5.71, 6.43

^a^3251 of subjects refused to answer the question about annual household income.

## Discussion

Based on the adjusted method, the prevalence of LBW and macrosomia was 5.15% and 7.35% for Chinese children under 6 years in 2013, respectively. The LBW rate was particularly high for children who were minority ethnicity, had less educated or older (maternal aged over 35 years) or younger (maternal aged under 20 years) mother, were in lower income household, or lived in west China or in rural areas. The macrosomia rate was particularly high for children who were Han ethnicity, were from higher income household, or lived in central or east China and urban areas, or whose mother had higher education degree or was older (maternal aged over 35 years).

In most cross-sectional studies, birth weight data were recalled by caregivers. Clustering of birth weight data in multiples of 500 g affects estimates of the incidence of low birth weight and macrosomia^15^. The birth weight data from maternal or other caregivers’ recall showed heaping to multiples of 500 g, which were influenced by their age, wealth index quintile, marital status and education^17,18^. As a result, some of the infants with birth weight exactly 2500 g will actually have lower or higher birth weight than 2500 g and those are exactly 4000 g will actually have lower or higher birth weight than 4000 g. This further underestimates or overestimates the prevalence of LBW and macrosomia^16^. In order to address the challenge of heaping in survey-based estimates of the prevalence of LBW, Boerma et al.^19^ and Blanc and Wardlaw^17^ developed a method to combine reported birth weights with additional variables, such as mothers’ perception of the child’s size at birth, which could increase the proportion classified as having low birth weight by 25%^17^. Another adjustment method of heaping issue is birth weight distribution method for health-facility records or caregivers’ reports, which was used to estimate national, regional, and worldwide estimates of low birthweight in 2015^2^. This method is also applicable to estimate the prevalence of macrosomia, which might provide more reliable estimates of the prevalence of LBW and macrosomia. Therefore, we fitted normal distributions to the birth weight data to produce more reliable estimates for the proportion of infants born with LBW and macrosomia.

Birth weight records were less clustering on certain digits than recalled birth weights^15,20^. Compared with the prevalence of LBW by the use of traditional calculation method (LBW (%) = total numbers of LBW/total numbers of surveyed children, 3.60%), the estimate using this heaping adjustment method (5.15%) was closer to that based on birth weight records in health facilities (5.36%)^11^. So does macrosomia in China. Compared with the prevalence of macrosomia with using traditional calculation method (5.30%), the adjusted estimate of 7.35% was closer to the estimate based on birth weight records in health facility (7.30%)^14^. Birth weight distribution adjustment method was more reliable than traditional calculation method for estimating prevalence of LBW and macrosomia based on recalled birth weight data or combined data of recalls and records.

Recent studies reported that LBW ranged from 2.77 to 6.1% in China^8–11^. The macrosomia rate varied between 6.5% and 10.44% in China^9,12–14^. Different types of data and estimate methods may contribute to the variation. For example, Hu^9^ estimated the prevalence of LBW (2.77% in 2006–2008) and macrosomia (10.44% in 2006–2008) by the use of traditional method based on recalled birth weight data. Yu et al.^8,13^ reported a prevalence of LBW and macrosomia of 4.60% and 6.50% respectively in 2006, Chen et al.^10^ reported a LBW rate of 6.10% and Li et al. reported a macrosomia rate of 7.30% in 2011 based on recorded data. In addition, different study areas and study population may partially explain the variation. One study was from 14 provinces in China^8,13^. Another study enrolled subjects from 5 provinces^9^. The third study included 12 cities and counties in southeast China^12^. The remaining studies were from 39 hospitals of 14 provinces^10,14^ or China’s National Maternal Near Miss Surveillance System (NMNMSS) in 441 health facilities^11^. Given that the lower hospital delivery rate in developing countries, especially in poor rural areas, and bias in the selection of delivery hospitals, estimates of the LBW and macrosomia rate from health-facility-based data are subject to bias and selection problems^17^. In 2014, more than 99.5% of newborns were born in hospital in China^21^. Although data on macrosomia was scarce, it was found that the prevalence of LBW varied in different class hospitals and the prevalence was higher in tertiary care hospital than that in secondary care hospital. Women with high-risk pregnancies or complications were transferred to high level hospital due to its advanced equipment and technology^10^. One of the advantages of survey data on birth weight is that they are from probability samples, which might reduce bias due to hospital selection and non-hospital delivery in developing countries. Therefore, nationally representative household surveys could improve the estimates of LBW and macrosomia rate with heaping adjustment.

Prevalence of LBW and macrosomia in developed countries were mainly based on health-facility recorded birth weight. Demographic and Health Survey (DHS) and Multiple Indicator Cluster Survey (MICS) collected birth weight recall data in developing countries and traditional estimates without adjustment of heaping issue may cause the prevalence of LBW and macrosomia substantially downwards^15–17^. The LBW rate of 5.15% in China was lower than the one in developed countries such as the United States (8.00%), the United Kingdom (7.00%) and Japan (9.60%)^2^. The macrosomia rate of 7.35% in China was lower than the one in the United States (8.23%)^22^ and England and Wales (8.80%)^23^, but much higher than Japan (0.90%)^24^ and Spain (0.65%)^25^.

Disparities existed across different regions of China in both LBW and macrosomia rate. The prevalence of LBW in economically underdeveloped western China was significantly higher than in central and eastern China and was also higher in rural areas than in urban areas. The prevalence of macrosomia was higher in relatively developed central and east China than in west China, and was also higher in urban areas than in rural areas. Previous studies in 2006^8,9^ and 2012–2014^11^ corroborated with our results. In addition, poor rural areas have higher macrosomia rate than medium and small cities and non-poor rural areas in our study, which may be related to higher percentage of missing birth weight records. Nearly 40% of 2059 ineligible or missing birth weight records was from poor rural areas.

Vulnerable subpopulation (mother aged under 20 years^26^ or over 35 years^27,28^, less educated mother^29,30^, low household income^31,32^, or minority) tend to have higher LBW rate. By contrast, subpopulation (maternal age over 35 years^33,34^, high educated mother^35^, high household income^36^ or Han ethnicity) tend to have higher macrosomia rate. This observation was consistent with previous studies’ findings in China^10,11,14^ and other countries^25,35,37–39^. Dietary, nutrition and educational interventions could significantly reduce LBW rate^40–43^, and diet and exercise interventions could reduce macrosomia rate^44,45^.

LBW and macrosomia has long been used as important public health indicators. Substantial heaping of birth weights occurs on particular values, usually values ending in 0 or 5. One obvious problem of this phenomenon is a resulting underestimate or overestimate of low birth weight or macrosomia and it would portray an overly optimistic or pessimistic picture of children’s and women’s health status. Therefore, accurate estimate of LBW and macrosomia is critical to reveal maternal and child nutrition and health status, to track progress towards national/global targets and to allocate health resource appropriately at the population level.

### Study limitations

This study had limitations that should be considered in the interpretation of the findings. There were 2059 birth weight records ineligible or missing. We found that the excluded subjects were more likely to be 3–5 years old, to live in rural areas, to be minority ethnicity, to have less educated mother, or to be in a lower income household. The finding that ineligible or missing birth weight records in rural areas accounts for about 70% of total 2059 excluded records and the proportion in poor rural areas was about 40% may affect the estimates of LBW and macrosomia by residential area. However, the proportion of infants with ineligible or missing birth weight accounted for no more than 6.0% of all study subjects and the gender and maternal age of subjects were similar for those included and excluded in the analysis. The impact is expected to be small for overall LBW or macrosomia estimates. Additionally, this is a cross-sectional study and birth weight data was collected by checking the birth certificate records or by caregivers’ recalls. Thus, to some extent, the results were affected by recall bias. However, the distribution adjustment we used to estimate the prevalence of LBW and macrosomia could reduce some bias due to the recall bias.

## Conclusion

After taking birth weight data heaping issue, the prevalence of LBW and macrosomia was 5.15% and 7.35% respectively, which are relatively lower than LBW estimates globally. Great attention needs to be paid to different areas and subpopulation groups (young or old mother, household income group) to control LBW or macrosomia. It would be needed to take potential misclassification of birth weight data arising from heaping into account for LBW or macrosomia estimation.

## Methods

### Subjects

Children under 6 years of ages who were free of birth defects and other health problems (such as cardiovascular diseases, chronic nephritis, tuberculosis, hepatitis, endemic diseases, chronic bronchitis etc.) were included, and they were categorized into 7 age groups (0–5, 6–11, 12–23, 24–35, 36–47, 48–59, and 60–71 months). The study was approved by the Ethics Review Board of the National Institute for Nutrition and Health, Chinese Center for Disease Control and Prevention (Number: 2013-018) and all methods were performed in accordance with the relevant guidelines and regulations^46^. Written informed consent was obtained from each child’s caregivers before the survey.

### Study design

The data of this study was from Chinese National Nutrition and Health Survey in 2013 (CNNHS 2013). Methods (including sample design, data collection and quality control procedures) have been previously described in detail^47^. Briefly, this was a cross-sectional study on the nutrition and health status of children under 6 years and lactating women. A multi-stage stratified cluster random sampling was used to select participants under 6 years of ages from 30 provinces, autonomous regions and municipalities in mainland China (Tibet autonomous region was not included in the survey). A total of 55 national representative survey sites were selected from the 2865 districts/counties/county-level cities using proportion to population size sampling method. Then three sub-district/townships were selected in each survey sites with using population size sampling method and three neighborhood/village committees were randomly selected in each sub-districts/townships. Overall, 495 neighborhood/village committees were selected in this study. Finally, 70 children (10 children in each age group, 0–5, 6–11, 12–23, 24–35, 36–47, 48–59, and 60–71 months) with equal number of girls and boys were selected in each neighborhood/village committees using a cluster sampling method. Totally, 34,650 records were collected in the survey.

### Outcomes and characteristics

A face-to-face interview was conducted to caregivers of children under 6 years. Birth weight data was collected by checking the birth certificate records or by caregivers’ recalls using a standard format and was recorded in grams in a questionnaire for children under 6 years. LBW is defined as a weight at birth < 2500 g and macrosomia is defined as a weight at birth > 4000 g. The household and individual’s demographic and socioeconomic information was collected using a household questionnaire, including infants’ age, gender, ethnicity, residential area, and maternal age, educational level, and household income level.

### Statistical analysis

One of the methodological issues when analyzing birth weight data is heaping of reported birth weights. Previous studies have found that respondents in national health surveys show a tendency to round birth weight to multiples of 100 and 500 g, for example 2500 g instead of 2485 g^15,17^. It is found that birth weight is approximately normally distributed under ideal conditions such as low-risk full-term singleton livebirths included in the WHO child growth standards^48^. In order to address the challenge of heaping, we adopted an adjustment method to fits birth weight data to a normal distribution to calculate the prevalence of LBW and macrosomia.

In summary, the proportion of low birth weight could be estimated in four steps^2^. First, we fitted two normal distributions of the data (full-term babies and preterm babies). Second, we calculated the LBW Z-score for each of the two normal distribution curves (full-term babies and preterm babies): Z_2500_ = (2500 g − Mean Birthweight)/SD_Birthweight_. Third, we calculated the percentage LBW for each of the two distribution curves: LBW (%) = probability of being below Z_2500_ (% area under the curve below Z_2500_). Finally, we calculated the overall LBW prevalence: Overall LBW (%) = (LBW (%)_term baby population_ × total number of term babies + LBW (%)_preterm baby population_ × total number of preterm babies)/total subjects. The proportion of macrosomia was calculated by the similar steps except a single normal distribution was fitted to the data. First, we fitted one normal distribution to the data. Second, we calculated the macrosomia Z-score for the normal distribution curve: Z_4000_ = (4000 g − Mean Birthweight)/SD_Birthweight_. Finally, we calculated the percentage macrosomia: macrosomia (%) = probability of being beyond Z_4000_ (% area beyond the curve of Z_4000_). The 95% confidence interval of LBW and macrosomia were also calculated by the same way.

Data were entered via a standardized data management platform and were cleaned for all variables. The prevalence is shown as percentage (95% confidence interval). We examined prevalence of LBW and macrosomia in the total population of and in sociodemographic subpopulations for children under 6 years. Student’s t-test and ANOVA was used to compare the means of each group and chi-square test to compare prevalence or proportion. All the data were analyzed using SAS 9.4 release (SAS Institute Inc., Cary, NC, USA). Statistical tests were two-sided and a P value below 0.05 was considered statistically significant.

## Supplementary Information

Supplementary Information.
