# Supplementary material for: Prevalence of low birth weight and macrosomia estimates based on heaping adjustment method in China
Source: Sci Rep. 2021 Jul 22;11:15016. doi: 10.1038/s41598-021-94375-2 (PMC8298412; doi:10.1038/s41598-021-94375-2)
Supplement: Supplementary file 1 — Supplementary Information. [file 41598_2021_94375_MOESM1_ESM.docx]

**Appendix. Supplementary data**

The included and excluded subjects were similar in gender and maternal age (**Table 1**). More children aged 3-5 years (60.9% vs 42.5%; P<0.0001), minority ethnicity (29.9% vs 13.1%; P<0.0001), with a lower educated mother (4.7% vs 10.9% with a college degree; P<0.0001), from lower income household (46.8% vs 34.8% with annual household income <10000 CNY; P=0.0008) or rural areas (71.9% vs 48.8%, P=0.0003) were seen in the excluded group.

**Table 1**. Demographic and socioeconomic characteristics of the subjects included and excluded.

| Characteristic | Children included(n=32276) | Children excluded(n=2059) ^a^ | P value |
| --- | --- | --- | --- |
|  | N (%) | N (%) |  |
| Age groups |  |  | P<0.0001 |
| 0-11 months | 27.1% (8746/32276) | 11.5% (236/2059) |  |
| 12-23 months | 16.2% (5234/32276) | 11.5% (236/2059) |  |
| 24-35 months | 14.2% (4567/32276) | 16.2% (334/2059) |  |
| 36-47 months | 14.8% (4787/32276) | 17.5% (361/2059) |  |
| 48-59 months | 14.4% (4652/32276) | 22.5% (464/2059) |  |
| 60-71 months | 13.3% (4290/32276) | 20.8% (428/2059) |  |
| Gender |  |  | P=0.5984 |
| Girl | 48.6% (15680/32276) | 48.0% (989/2059) |  |
| Boy | 51.4% (16596/32276) | 52.0% (1070/2059) |  |
| Residential area |  |  | P<0.0001 |
| Urban-metropolis | 22.6% (7299/32276) | 14.4% (296/2059) |  |
| Urban-middle or small cities | 28.6% (9220/32276) | 13.7% (283/2059) |  |
| Rural-general areas | 32.7% (10565/32276) | 32.0% (658/2059) |  |
| Rural-poor areas | 16.1% (5192/32276) | 39.9% (822/2059) |  |
| Ethnicity ^b^ |  |  | P=0.0002 |
| Han | 86.9% (28037/32267) | 70.1% (1443/2059) |  |
| Minority | 13.1% (4230/32267) | 29.9% (616/2059) |  |
| Maternal age groups ^c^ |  |  | P=0.1548 |
| <20 years | 1.2% (355/30421) | 1.2% (20/1709) |  |
| 20-25 years | 25.1% (7627/30421) | 24.2% (414/1709) |  |
| 26-30 years | 37.2% (11321/30421) | 35.9% (613/1709) |  |
| 31-35 years | 24.1% (7333/30421) | 22.9% (392/1709) |  |
| >35 years | 12.4% (3785/30421) | 15.8% (270/1709) |  |
| Maternal education ^d^ |  |  | P<0.0001 |
| Primary or below | 12.6% (3841/30599) | 23.6% (413/1748) |  |
| Junior high school | 47.2% (14450/30599) | 53.0% (927/1748) |  |
| High school | 18.2% (5569/30599) | 12.6% (220/1748) |  |
| Associate's degree | 11.1% (3392/30599) | 6.1% (106/1748) |  |
| Bachelor's degree or above | 10.9% (3347/30599) | 4.7% (82/1748) |  |
| Annual household income (per capital CNY) ^e^ |  |  | P=0.0008 |
| <10000 | 34.8% (11242/32261) | 46.8% (963/2058) |  |
| 10000-19999 | 27.6% (8888/32261) | 24.5% (504/2058) |  |
| ≥20000 | 27.5% (8880/32261) | 17.8% (366/2058) |  |
| Refuse to response | 10.1% (3251/32261) | 10.9% (225/2058) |  |

^a^ The 2026 missing birth weight records and 33 outliers were excluded;

^b^ The ethnic information for 9 subjects was missing;

^c^ Information on maternal age was missing in 1,855 subjects;

^d^ Information on the maternal educational level was missing in 1,677 subjects;

^e^ Information on the annual household income was missing in 15 subjects.

**Age - gender specific birth weight distribution**

The mean birth weight among children under 6 years was 3294.9 ± 484.8 g (**Table 2**). The 10^th^ percentile of birthweight was 2700 g. Children aged 36-47 months had lower birth weight than those aged 0-5 months (t=4.23, p=0.0005), 6-11 months (t=3.35, p=0.0170), and 12-23 months (t=3.31, p=0.0199). Birth weight differs by gender. Boys’ birth weight are higher than girls’ (3335.55 ± 491.31 vs 3251.89 ± 473.95, t=15.57, P<0.0001).

**Table2**. Birth weight distribution among Children under 6 years by gender and age groups, China, 2013. (grams)

| Characteristics | n | Mean ± SD | P5 | P10 | P25 | P50 | P75 | P90 | P95 |
| --- | --- | --- | --- | --- | --- | --- | --- | --- | --- |
| Age (month) |  |  |  |  |  |  |  |  |  |
| Total | 32276 | 3294.91 ± 484.76 | 2500 | 2700 | 3000 | 3300 | 3600 | 3900 | 4050 |
| 0-5 | 4293 | 3318.19 ± 478.79 | 2600 | 2800 | 3000 | 3300 | 3600 | 3900 | 4100 |
| 6-11 | 4453 | 3308.94 ± 486.72 | 2500 | 2750 | 3000 | 3300 | 3600 | 3900 | 4060 |
| 12-23 | 5234 | 3307.19 ± 495.21 | 2500 | 2700 | 3000 | 3300 | 3600 | 3900 | 4100 |
| 24-35 | 4567 | 3278.69 ± 483.07 | 2500 | 2700 | 3000 | 3250 | 3560 | 3900 | 4000 |
| 36-47 | 4787 | 3275.15 ± 479.07 | 2500 | 2700 | 3000 | 3250 | 3520 | 3900 | 4050 |
| 48-59 | 4652 | 3287.18 ± 485.36 | 2500 | 2700 | 3000 | 3250 | 3600 | 3900 | 4100 |
| 60-71 | 4290 | 3289.75 ± 481.74 | 2500 | 2700 | 3000 | 3250 | 3600 | 3900 | 4010 |
| F/P value |  | F=5.29, P<0.0001 |  |  |  |  |  |  |  |
| Gender |  |  |  |  |  |  |  |  |  |
| Boys | 16596 | 3335.55 ± 491.31 | 2515 | 2800 | 3000 | 3300 | 3600 | 3950 | 4100 |
| Girls | 15680 | 3251.89 ± 473.95 | 2500 | 2700 | 3000 | 3200 | 3500 | 3800 | 4000 |
| t/P value |  | t=15.57, P<0.0001 |  |  |  |  |  |  |  |
